# Preventable infant deaths, lone births and lack of registration in Mexican indigenous communities: health care services and the *afterlife of colonialism*

**DOI:** 10.1080/13557858.2018.1481496

**Published:** 2018-06-19

**Authors:** Jennie Gamlin, David Osrin

**Affiliations:** Institute for Global Health, University College London, London, UK

**Keywords:** Indigenous, Mexico, maternal health, stillbirth, infant mortality

## Abstract

Mexico’s indigenous communities continue to experience higher levels of mortality and poorer access to health care services than non-indigenous regions, a pattern that is repeated across the globe. We conducted a two-year ethnographic study of pregnancies and childbirth in an indigenous Wixárika community to explore the structural causes of this excess mortality. In the process we also identified major differences between official infant mortality rates, and the numbers of infants born to women in our sample who did not survive.

We interviewed 67 women during pregnancy and followed-up after the birth of their child. At baseline, socio-demographic data was collected as well as information regarding birthing intentions. In depth-interviews and semi-structured interviews were conducted with 62 of these women after the birth of their child, using a checklist of questions. Women were asked about choices regarding, and experiences of childbirth.

Of the 62 women we interviewed at follow-up 33 gave birth at home without skilled attendance and five gave birth completely alone in their homes. Five neonates died during labour or the perinatal period. Concerns about human resources, the structure of service delivery and unwanted interventions during childbirth all appear to contribute to the low institutional childbirth rate. Our data also suggests a low rate of death registration, with the custom of burying infants where they die. This excess mortality, occurring in the context of unnecessary lone and unassisted childbirth are structurally generated forms of violence.

## Background

Globally, neonatal mortality accounted for 42% of under-five mortality in 2013, an increase from 37% in 1990 (Wang et al. 2014). A systematic review of childbirth care packages estimated that skilled birth attendance could reduce neonatal mortality by 25% (Lee et al. 2011), while basic and comprehensive emergency obstetric care could reduce neonatal deaths by 40% and 85%, respectively. A recent study of access to antenatal care in six Mesoamerican (Mexico and Central America) countries found that many women in the poorest regions were still not receiving adequate antenatal care (Mokdad et al. 2015), while ethnic differences in facility childbirth rates were 15.2% vs. 41.5% in Guatemala and 29.1% vs. 73.9% in Mexico for indigenous and non indigenous women (Colombara et al. 2016). Data from across the globe coincides in the fact that health outcomes and skilled attendance at birth are worse among indigenous populations than their national average (Gracey and King 2009). It is also known that geographical isolation, lack of disaggregation by ethnic group and inadequate surveillance data compromise the availability and accuracy of data (Valeggia and Snodgrass 2015), suggesting that these figures may underestimate true rates. We were unable to identify data that compared stillbirths for these populations but would expect this to reflect similar inequalities.

Mexico’s indigenous people continue to experience higher levels of poverty, lower school enrolment and worse health outcomes than their mestizo counterparts. At a national level life expectancy at birth has reached 77 years, but this figure masks inequalities, as life expectancy for the indigenous population is 69 years (UNDP 2010). The divide is greater for infant mortality. Using the most recent comparative data, the Infant Mortality Rate (IMR) was 14 per 1000 live births in the non-indigenous population, but 23 per 1000 in indigenous populations (UNDP 2010). Although the national under-five mortality rate (U5MR) is now only slightly above the fourth Millennium Development Goal target of 15 per 1000 (WHO 2013), Mexican indigenous people are far from these goals. The U5MR was 76 in the predominantly indigenous municipality of Mezquitic in which our study was conducted (CONAPO 2005), reflecting the global tendency for worse health outcomes among indigenous than non-indigenous people within the same nations (Gracey and King 2009; King, Smith, and Gracey 2009; Anderson et al. 2016).

Using the description given by Montenegro and Stephens (2006), we define indigeneity as the identity of the *original* inhabitants of an area: ‘the descendants of the original inhabitants who were colonised, and those who live in an indigenous way and are accepted by the Indigenous community’ (p1859). Key to this description is the relationship with colonisation, and the fact that indigenous people continue to live under state and political systems that were imposed upon them, and originate from different and dominant ethnic, cultural and social structures. Approximately 10 million of Mexico’s nearly 120 million inhabitants are indigenous, belonging to one of 67 separate ethnic groups, distinguished by language, customs and belief and accounting for the largest number of indigenous people in any one country of the Americas (Montenegro and Stephens 2006). First nations communities throughout the Americas share similar histories of massacre, ethnocide, colonisation, and differential degrees of conformity and autonomy within nation states. Understanding the consequences of this history and its continued presence in social and health policies and interventions is important if we are to unravel the structural determinants of excess morbidity and mortality among indigenous groups. Research about health inequalities in Australia has revealed a historical relationship between colonialism and indigenous health, with negative factors such as on-going racism, a self-perception of inferiority, historical grief and loss contributing to poorer health outcomes among aboriginal groups (Paradies 2016; Griffiths et al. 2016). An anthropological review study of indigenous health globally also highlights the ‘devastating effects of colonization, the loss of ancestral land, language and cultural barriers for access to healthcare’ (Valeggia and Snodgrass 2015, 117). These are aspects of indigenous health and lives would not be easily identified using epidemiological methods (Stephens et al. 2006), or may in fact be entirely ignored. In this paper we will refer to this relationship between indigenous communities and the state as the *afterlife of colonialism*: the on-going impact of a colonial legacy that continues to reduce life chances, leads to preventable mortality and is present in everyday relationships between people and institutions.

The Mexican *Seguro Popular* health insurance system was established in 2004 to provide free healthcare in Ministry of Health institutions. Although this programme brings near universal health insurance to Mexico, it is also contradictory. Mexico’s indigenous people and all pregnant women were already entitled to these services free of charge, but now they must register with the Seguro Popular to gain access. In spite of near universal and free cover, indigenous people continue to access healthcare less frequently. Only 76% of indigenous compared to 94% of non-indigenous women giving birth in health facilities (Leyva-Flores et al. 2013). Finally, service provision in many communities and for many families is linked to a conditional cash transfer scheme. The *Prospera* cash transfer programme that operates in poor communities requires affiliates to attend exercise sessions and health talks, as well as to ensure their children’s school attendance. In the region where the study was conducted the conditionality of *Prospera* is controlled by local health clinics, which record attendance and apply sanctions. This ensures that clinic staffs are able to exert a level of authority over patient’s lives that can in itself become a source of conflict.

Although 93% of births in Mexico were assisted by a skilled birth attendant (SBA) in 2014, and 88% of births were institutional, only 30% of births to women in Mezquitic, the predominantly indigenous municipality where this study was conducted, were attended by a SBA and 55% were home births (INEGI 2015a). Only six neonatal deaths were reported in 2014, and there was wide variation in total birth registration, with 1639 registered in 2010 and 1271 in 2014 (INEGI 2015b). Based on these data, the extended perinatal mortality rate (stillbirths and neonatal deaths per 1000 births) for the municipality was 4 per 1000 (95% confidence interval 2,10). This low estimate of mortality prompted us to examine our ethnographic data from the perspective of structural determinants of poor maternal and child health outcomes and to explore the system of birth and death registration.

Structural determinants of health are factors influencing wellbeing, which are ‘embedded in the political and economic organisation of our social world’ (Farmer et al. 2006, 1686). They are historically constructed institutions such as gender (see for example Risman 2004 and Connell 1987), race and class (Farmer et al. 2006; Farmer 2005; Bakan and Dua 2014), that regulate social status, privilege and access to resources, and through this, influence interactions with and between people. Quality of care, language and cultural barriers, poverty, unwanted or inappropriate treatment have all been documented as structural barriers to indigenous care seeking in Mexico (WHO 2016; Smith-Oka 2009; Zacher Dixon 2015). We also know that inequality in the structure of gender, makes pregnant women particularly vulnerable to exclusion, because they are less likely to control their own financial resources and are vulnerable to abuse within and outside the home (Sesia, Zentella, and Ruiz 2007; Freyermuth Enciso and Argüello Avendaño 2010; González Montes and Valdez Santiago 2008).

In this paper we will extend analyses beyond these issues to explore how on-going frictions between indigenous communities and the state, which are a consequence of colonial history (Stephens et al. 2006; Montenegro and Stephens 2006) continue to operate via the health system, leading to preventable infant mortality and stillbirths. To do this, we interrogate data from our ethnographic study that pertains to the *structure of service provision*. We do this from a theoretical standpoint of structural violence, a position that seeks to understand how social structures make certain people more vulnerable to morbidity and mortality (Farmer et al. 2006).

## Methods

Gathering epidemiological information on pregnancies and births in indigenous regions is frequently complicated by accessibility, difficulty working in minority languages and well founded historical distrust (Stephens et al. 2006). Anthropologists have led work to improve understanding of indigenous people, and are well positioned to illuminate the structural and historical factors that shape illness and mortality in first nations communities (Valeggia and Snodgrass 2015; Pfeiffer and Nichter 2008). For these reasons and so that we could focus on gaining a rich and multi-layered understanding of women’s experiences of pregnancy and childbirth, we used ethnographic methods to explore the determinants of preventable maternal and infant deaths in Wixárika communities.

### Population and setting

The Wixárika ethnic group live in the Sierra Madre mountains, on the northern tip of Jalisco state. In common with other first nations communities across the globe, Wixárika people maintain a particularly strong relationship with the natural and supernatural world and this continues to influence their understanding of illness causality (Gamlin 2016). Unlike southern Mexican ethnic groups the Wixárika, do not have a system of traditional birth attendants and it is common for women to birth alone or supported by a family member (Chopel 2014). Our study took place in the 14 Wixárika towns and villages that make up the community of Tuapurie, municipality of Mezquitic, with a population of approximately 2500. 77% of Mezquitic’s population of 18084 are indigenous (INEGI 2015a). About half of the population of Tuapurie live in two vehicle-accessible towns, each with a rural health clinic between two and five hours from the nearest hospital, the remainder live in hamlets up to a further six hours on foot.

### Design

Pregnancy (baseline) and postpartum (follow-on) interviews, key informant and stakeholder interviews, and focus groups were conducted as part of an ethnographic study of the structural determinants of maternal and child health in Wixárika communities. Structured pregnancy interviews asked about socio-demographics, gender equality, previous pregnancies, antenatal care, and childbirth intentions. We used semi-structured interviews with a question guide after delivery to ask about the birth attendance and outcomes. The interview guide included prompts for women to report on choices regarding childbirth, interactions with health professionals, and satisfaction with institutional care. For the 24 month duration of the study we also gathered observational data on clinic services and general living arrangements and recorded these in a field diary. Ethnography is usually an inductive methodology through which the researcher introduces new lines of inquiry as they arise within the research process (Holmes and Castañeda 2014). During the course of our data collection we identified high numbers of neonatal deaths that did not align with official vital registration data. This led us to conduct further interviews on the topic of birth and death registration.We report on the findings of these interviews according to the Consolidated Criteria for Reporting Qualitative studies, COREQ (Tong, Sainsbury, and Craig 2007).

### Procedures

We used ethnographic methods to explore the structural origins of maternal and infant morbidity and mortality by analysing interactions between people and between people and institutions. The project was discussed with key community members and at local village meetings before being presented to the wider community at their general assembly meeting, where it was officially approved. Ethical approval was granted by the University College London Ethics committee. Interviewees were initially asked to participate at community meetings, and then approached individually. Consent was given verbally.

### Research team and reflexivity

The principal investigator was a medical anthropologist with expertise health inequalities. She had worked in the participating communities since 2008. Eight female bilingual (Wixárika/Spanish) women were trained as interviewers using pilot questionnaires with non-study women, and the translation-back translation method was used to ensure shared understanding in Wixárika or Spanish. The interviewers were all residents of Tuapurie and worked part-time on the study for two years. They conducted pregnancy interviews. The principal investigator conducted postpartum interviews with translation assistance.

### Participant selection

Local village meetings were used as a platform for discussing the project with the community and to invite women to participate. Interviewers then made visits to the homes of pregnant women, identified by word of mouth, and asked them personally to participate. 67 women were interviewed at baseline and there were eight refusals, with most citing a reluctance to share personal information. Using the most recent census data (INEGI 2015a), we estimate that we interviewed approximately one third (35%) of all women who gave birth in Tuapurie between January and December 2015, and approximately 5% of all pregnant women in the municipality of Mezquitic.

### Limitations

We were interested in interviewing all women who were pregnant and willing to participate, but due to the practical difficulties of finding women from distant valley communities in their homes, we expect this group is underrepresented. Due to migratory practices and the farming and cattle ranching lifestyles of Wixárika families, we were not able to follow-up with all of the women who participated at baseline. In these cases we spoke to a relative who was present at the time of the birth. While this enabled an almost complete set of before and after data with regards to pregnancy and birth outcomes, these data are limited in depth and detail.

### Data collection

Interviewers were encouraged to use their local knowledge to conduct interviews when the husband or other male relatives were not present, but this was not always possible. The pregnancy (baseline) questionnaire asked participants about antenatal care, birthing intentions, fertility history and household division of tasks. Women were followed up in their homes after childbirth with audio-recorded semi-structured interviews lasting between 15 min and one hour. Interview data were triangulated with observations recorded in a field diary and informal interviews with health providers and community elders. Findings were presented to women at community meetings and events.

### Data analysis

Methods that focus on ensuring individual and community voices are heard including processes of gaining trust, gathering birth stories and verbal autopsies, have been successfully used to extract data on births and deaths in complex settings (Briggs 1986; Scheper-Hughes 1992). To obtain results on birth outcomes numerical data were extracted from in-depth interviews and entered into an Excel spread sheet (Microsoft Corporation) for analysis. Interviews were transcribed directly into Spanish and ethnographic data were analysed using N-Vivo (version 8). We conducted an inductive thematic analysis of the qualitative data, which included interview transcripts and field diary notes, first identifying general nodes (themes) and then adding a second layer of coding to each node. Data were coded separately in Spanish by one bilingual (Wixárika-Spanish) and one Spanish-speaking research assistant, and checked for accuracy by a third person. Emergent themes included social and cultural issues relating to maternal and child health, Wixárika traditions and customs, gender inequalities, and health systems issues. In this paper we focus on data pertaining to health systems and registration.

## Findings

### Health facilities and services

There were two rural health clinics (‘A’ and ‘B’) in our study site, which together served all fourteen towns and villages in Tuapurie. The clinics provide basic healthcare under a single Ministry of Health structure of service provision, where staff work 20 consecutive days followed by ten days off. Each clinic should have been staffed by a family doctor and nurse, from days 11–30 of each month. A trainee doctor was in attendance from days 20-10, staffing the clinic alone for the first ten days of each month. However, there were numerous uncertainties on the part of providers and patients about lack of consumables and medications, transportation, staffing levels and turnaround. All of these factors affected clinical quality of care and client perception of care in general.

All non-local staff live on-site, and although clinics are only open during the daytime, doctors are ‘on call’ 24 h a day for emergencies. However, there were important differences between the two clinics in terms of both staffing and facilities. The clinic in community ‘B’ had two separate consultation rooms, a birthing or examination room, separate recovery room and a reception/waiting area. In contrast, the clinic in community ‘A’ opened directly into the one consultation room, which providers and clients had to walk though to reach the toilet or examination room, and to enter or leave the clinic.

Clinics were clean and stocked basic medication for common ailments and problems specific to the region, such as scorpion anti-venom. All pregnancy related sampling took place at the nearest hospital as clinics did not have the facilities to store or transport biological samples safely. This meant that pregnant women were often not tested at any time during their pregnancy and ultrasound examinations were not routine. The basic facilities and distance to a hospital meant that, officially, clinic staff should not attend births. In practice, and as we see from birth data gathered during this study (see Table 1), many women did give birth in the clinics. Women with high-risk pregnancies were referred to the nearest hospital and would ideally stay in municipal hostels for the final weeks of pregnancy, although only one woman in our sample chose this option.

**Table 1.** Details of birth attendance, based on 62 paired interviews.

	Community A	Community B	Mean age of mother	Primi-gravidae	Mean previous live births
Lone birth	2	3	27.2	0	3.8
Home birth with help	14	14	28.3	5	3.5
Clinic birth	4	17	23.4	7	2.09
Hospital birth	3	5	23.1	3	2.14
Total/mean	23	39	24.5	15	3.1

Transport and communications in the highlands are poor and clinics did not have telephones or internet. Public transport to and from the municipal town and hospital is prohibitively expensive for many families. An overnight hospital stay would incur additional costs for food, bus fares for other family members, and in some cases hotel accommodation. A return bus service usually ran from community B every day, while the bus from community A ran three times a week and returned the following day. The slow and uncomfortable journey to both communities is along a stony mountain track which can become impassable during rainy season.

### Births and pregnancy outcomes

We carried out a total of 67 pregnancy interviews and 62 post delivery, two women miscarried and 3 were unavailable for follow-on interviews. The mean age of women at first interview was 24.5 years (range 13-39). Women had a mean 3.1 children (range 1-9) and 15 were primigravid.

Less than half of women (47%) gave birth with a skilled birth attendant. Those who gave birth at home did so either alone or with help of their spouse or a relative. Five women lost their babies at term, during or shortly after birth. Three of these had home births, assisted by a family member, one baby was born in a clinic and died within a week, and another was born and then died in the hospital.

### Structural weaknesses in service provision

#### Quality of care in clinics

We found mixed responses to the quality of care and perceptions of care within and between communities. Mostly women who chose to give birth in a clinic did so because they had confidence in the staff on duty and were tolerant of medical interventions. None of the women reported verbal abuse from staff in the community clinics but they did describe invasive and unwanted routine practices and a lack of understanding about what was happening to them. One of these was the routine use of episiotomy, to which they did not consent or of which they were unaware. As H (22 years) said, ‘When Leandro’s sister was born they cut me, I felt like kicking them in’. In fact there was a generalised misunderstanding of what an episiotomy was, L (20 years), who gave birth in a hospital, had been told she was sent there because she was overdue and might need a caesarean birth. She tells us; ‘They sent me [to the hospital] because I was more than nine months and so that they could do a caesarean’. It in fact turned out that she had been given an episiotomy, but she had left the hospital thinking it had been a caesarean. Like many others in both clinics and hospitals she was also given an injection but had no idea what it was.

NS (17 years) reiterated this lack of understanding when she described her experience of childbirth in one of the clinics:

Well, when I went there at 10pm they made me stay. They took my clothes off me, made me lie down and gave me injections. I was really embarrassed but what could I do? Then they put me on a drip … I don’t know what for, they just gave me an injection for the pain and a drip, but I never knew what it was for.

During the study, three different doctors were posted to clinic A, one of whom was accused of insisting on a series of measures that were found offensive and provocative by members of the community. R (31 years), who was also a translator at the clinic, said,

She told us she was in charge and we had to do what she said. We were told we had to make an appointment the day before, but it takes some women hours to walk to the clinic; then if we arrive late she won’t see us and we have to come back another day. She also told us we would lose our Prospera money if they don’t come for the exercise sessions, even if they have to walk from valley communities [at 2–4 h walking distance].

The case of this particular doctor led the community to submit a series of official complaints to Ministry of Health officials at municipal and state level, including a request for the doctor to be replaced.

#### ‘There’s never anyone there’. Human resources, service provision and home births

Many of the women who gave birth at home did so because they where sceptical about the availability of staff or anticipated poor and invasive treatment. Although the official line from doctors and nurses was that *there is always someone in the clinic*, this was not the impression that many interviewees gave. It was common knowledge that the family doctor was only in the clinic from 11th to 30th of each month, with the first 10 days of the covered by a trainee. There was also a high turnover of trainees, often leaving gaps of several months when there was no-one to cover the family doctor’s absence and leaving the impression that there was no-on there. H (22 years), who gave birth to her second baby in the kitchen of her aunt’s house while she was on her way to the hospital, said that she chose not to go to the URM because ‘my son was born in the [doctor’s] holidays and I thought the doctors wouldn’t be there … he was born on the 10th and they arrive on the 11th.’ This concern about not being received was coupled with a previous unhappy experience of birthing with her ankles ‘strapped high above her head’*.* H was fortunate: her baby was born well, but her sister-in-law had lost her baby two months before in the same kitchen. D’s baby was born in breech position, also on the 10th day of the month, and did not survive. Her sister said, ‘It took about three hours, I just listened to her screaming and screaming  …  afterwards they took her to the clinic, but the baby died because he came feet first.’

There were considerable differences between the two clinics, not only in terms of facilities and distance to the regional hospital, but service provision and staff turnover was also considerably worse in community A. Over the course of this research project the family doctor changed three times and there was a generalised lack of trust in clinic staff. With each change of staff the clinic would lack either a family doctor or a trainee doctor for weeks or months, leaving the impression that there was *never anyone there*, a statement made on multiple occasions.

In contrast, the doctor in clinic B had earned herself community trust, was well liked, and women were confident that she would attend them from 11th to 30th of each month. The clinic also retained its trainee doctors. In contrast, there were long periods when only a nurse and health promoter staffed the clinic in community A. Notable contradictions in service provision were also evident. While the family doctor (when available) was willing to attend births, in spite of complaining that ‘we often have no gloves, no oxytocin, no vitamin K and no serum’, other staff were not. The nurse in clinic A explained to us that ‘If a women comes in labour I tell her to go [to the nearest hospital]; we don’t attend births here unless we absolutely have to,’ while the trainee doctor in the same clinic assured me that women were not interested in seeking antenatal care or giving birth at the clinic. ‘They don’t want to come’ he explained. ‘It is their custom to give birth at home, and anyway we don’t have the materials to attend births … but there is always someone here’. He said that he felt the clinic gave a good service and if women did not come it was because of their tradition for home birthing.

Salaries of doctors in the two clinics also differed. Doctors in clinic A received approximately USD $400 and were hired on 12 month contracts. In contrast the doctor in clinic B was on a permanent contract and paid around double this amount. The family doctor in community A was vocal about the fact that she only intended to be there for one year, ‘as a personal challenge’, and explained that doctors leave as soon as there are offered a better contract. In addition to pointing out the lack of supplies, she deferred responsibility for problems to a higher level, explaining that she had arrived to find a generalised lack of order and that ‘the authorities think there is no problem’.

### Birth and death registration

Of the five infants from this study who died in the perinatal period, three were born to women in their homes, at a considerable distance on foot from the clinic. None of these were registered as births or deaths. C (20 years) who lived two hours on foot from the clinic, said, ‘Once my labour had started it was already too late.’ C’s baby became ill shortly after the birth when she and her husband were on their way to a ceremony. There they consulted a local shaman, but did not return to the clinic and buried their baby in the small hamlet where he died.

A further concern that emerged clearly from our data was the lack of birth or death registration. The community registrar should record births, deaths and marriages, but couples rarely legally married and deaths were rarely registered. Due to the requirements for school enrolment and the *Prospera* welfare programme, most births are now eventually registered, although it is common for this to be delayed until a child is several months or years old. A key informant interview with the registry clerk revealed that ‘ …  there were about ten deaths last year in this town, but only three were registered. There was only one baby death registered’. On further questioning we confirmed that this one infant had not been included in our study. The nurse at clinic B confirmed that registration of births and deaths was not enforced, and it also became clear that doctors were not always aware of stillbirths, perinatal or infant deaths in the community, even in cases where the birth took place in the clinic. This was the case for a baby who died at one week of age. Her grandmother said that

things became worse after she was vaccinated. When we took her to the healer [shaman] he told us [the baby] was very ill. Maybe we could have done something, but as we had no money we didn’t take her anywhere because, well, what would happen if they sent us [to a hospital] far away, what are we going to do then if we have no money. Anyway, in her last days the baby didn’t look like she would survive even if we took her to the clinic.

Upon follow-up nine months later, we were able to confirm that the baby had not been registered or reported to the clinic. Several of the deaths to women who were in our study appear to have been intrapartum stillbirths. These are also unaccounted for in either ministry of health or vital registration data.

## Discussion and conclusions

Physical distance and limited connectivity, cultural and language barriers are the geocultural challenges to healthcare access faced by Mexican indigenous communities, and they have been well documented in ethnographic research (Farmer et al. 2006; Holmes 2012). However, this cultural and physical isolation must be denaturalised so that it is not viewed as an accident of nature, brought on by indigenous people’s lifestyle choice or the geography of their homelands. Mexico is an extremely diverse country that has overcome natural barriers to connectivity such as the lakebed and earthquake prone zone on which Mexico City was built, and the mountainous regions of central Mexico that went on to form affluent silver mining towns and cities. The state’s *avoidable* failure to provide for and protect indigenous and rural populations who are in various ways isolated, is therefore a form of structural violence in itself: ‘ … arrangements that put individuals and populations in harm’s way. The arrangements are structural because they are embedded in the political and economic organisation of our social world; they are violent because they cause injury to people’ (Farmer et al. 2006, e449).

### Structural weaknesses leading to infant mortality

Alongside the naturalisation of geocultural neglect that is a central aspect of the *afterlife of colonialism,* analyses of the structural determinants of maternal mortality in Chiapas highlight the role of racism, poverty and gender inequality, social inequalities that lead to higher mortality among indigenous populations (González Montes and Valdez Santiago 2008; Freyermuth Enciso and Argüello Avendaño 2010). Ethnographic studies with indigenous communities in Oaxaca state, which together with Chiapas and Guerrero shares the double vulnerability of widespread extreme poverty and a high proportion of indigeneity, describe structural and gender violence as well as mistreatment and abuse by healthcare providers, operating as barriers to care seeking and service uptake (Sesia, Zentella, and Ruiz 2007; Berrio Palomo 2014). Our data extend structural analyses of gender, race, and poverty by exploring how health systems and the structure of service delivery add a further layer of structural violence, one that is directly related to the hierarchical relationship between indigenous communities and the state. Our central finding was that the structure of healthcare delivery in Wixárika indigenous communities, combined with an unnecessary level of medicalisation including routine episiotomies, childbirth using stirrups and the use of oxytocin during a normal labour appeared to deter women from seeking skilled care during pregnancy, childbirth, and the postpartum period, leading to preventable stillbirths and perinatal deaths. These health system weaknesses are compounded by a lack of connectivity as there are no phone lines or Internet connections with regional hospitals, and roads are unpaved, making them at times both unsafe and impassable. Short-term contracts and low wages ensure a high staff turnover, while the monthly structure of service has generated a community wide uncertainty about the availability of all types of medical care, including birth attendance. Medical staff do not appear to share these concerns. On the contrary, there appears to be a belief that women give birth alone, and risk their lives, because it is their tradition. While at a structural level, the Ministry of health has been unresponsive to the lack of medical resources available to clinics. These service delivery issues are compounded by a historical distrust and underlying tension between indigenous communities and the state, evidenced through hierarchies established by clinic doctors leading to conflict between clinic staff and communities. A recent study of institutional childbirth and satisfaction among Mexican and Guatemalan indigenous and poor women, found that being accompanied by a community health worker and facility staff speaking their language, were the primary determinants of satisfaction (Colombara et al. 2016). We know that access to skilled childbirth is a key determinant of maternal and infant morbidity and mortality (Aminu 2014), yet neither of these resources, which could potentially have increased service uptake, were available to women in this study.

### Registration

A second structural weakness is the likelihood that deaths and stillbirths are underreported, or not reported at all, due to a weak vital registration system. Using a representative sample of 101 Mexican municipalities with the highest levels of marginalisation, Hernandez et al. estimated that infant deaths had been underreported by 23%, while a study in Guerrero state suggested underreporting by 69%–73% (Tomé, Reyes, and Piña 1997; Hernández et al. 2012). According to official data for 2014, there were six stillbirths or neonatal deaths after 1271 births in the municipality of Mezquitic (INEGI 2015b). 2012 data describe two neonatal deaths, while in 2010 no neonatal deaths were registered. A rough comparison of INEGI data on neonatal deaths in the municipality and our incidental findings suggests that official registration is underreporting infant mortality rates. We estimate that our respondents accounted for approximately 5% of annual municipal births. Even if the highland context was only representative of approximately 75% of the municipal population (25% live in non-indigenous foothills villages within one hour of a hospital), we can say that the official rate was very low and that it is likely that official figures under-count infant births and deaths in the region.

Our ethnographic findings suggest that significant proportions of stillbirths and early new-born deaths of infants born at home remain unregistered, an important weakness in both health system and vital registration systems. We are concerned that this problem may be endemic in Mexico’s indigenous populations, a hypothesis that at requires testing. Conclusive findings would have a wide-ranging impact not only on how data are reported and collected but also on addressing the causes of infant mortality.

A recent Lancet series on indigenous health highlighted the need to recognise that

Not all Indigenous populations in the world have endured precisely the same forms of violence, but one common factor has been the experience of enclosure in a national state that defines its identity and priorities in ways that ignore, marginalise, denigrate, or actively suppress Indigenous communities. (Kirmayer and Brass 2016, 105–106)

We have identified some of the nuanced ways in which national structures such as health systems can marginalise or suppress indigenous communities, so that women feel unable to seek health care when it is needed. Documents written in a foreign language continue to be a source of distrust for the Wixárika, as for other Mexican indigenous groups. Poor vital registration is a case in point that symbolises indigenous rejection of a national system to retain control of populations. Here we argue for the importance of vital registration in identifying both rates and causes of infant death. These intertwined issues need to be understood as part of the afterlife of colonialism.

A geopolitical history of ethnocide and colonialism has ensured that Wixárika people share characteristics with indigenous populations throughout the Americas, and particularly with those in the culturally bound Mesoamerican region (Montenegro and Stephens 2006). The Salud Mesoamérica initiative has documented considerably lower rates of antenatal care and skilled delivery in Guatemala, a country that is 40% indigenous, where only 15.2% of indigenous compared to 41.5% of non indigenous women give birth in facilities (Colombara et al. 2016). Further south, Perú and Bolivia face similar inequalities (Hall and Patrinos 2005), suggesting that our findings will be relevant to a wide geographical region where the overlapping vulnerabilities of indigeneity, poverty and inadequate service provision are widespread.

A recent WHO press release (2016) describes investment in health employment as key to attaining the Sustainable Development Goals. Our findings suggest that investing in local service provision could yield disproportionate dividends for perinatal survival by increasing skilled attendance at birth. Accurate vital registration is also needed if we are to know whether the health system is working effectively in marginalised populations. These are achievable targets for a middle-income country and there should be no further delay in addressing them.

In addition to responding to weaknesses in local and national health systems, it is vital that the global health community and nation states recognise and seek to redress the on-going forms of violence against indigenous ethnic groups, often expressed as racism, neglect, denigration or marginalisation, which are the result of their continued experience of enclosure in a modern national state. This afterlife of colonialism that comes in the form of structural and political oppression leading to reduced life chances, is not a natural feature of our social world and it requires concerted political will to bring about the much needed economic, social and political changes.

## References

[CIT0001] Aminu, M. 2014. “Reducing Neonatal Mortality through Skilled Birth Attendance.” *The Lancet Global Health Blog*, May 6.

[CIT0002] Anderson, I., B. Robson, M. Connolly, F. Al-Yaman, E. Bjertness, A. King, M. Tynan, et al. 2016. “Indigenous and Tribal Peoples’ Health (*The Lancet*–Lowitja Institute Global Collaboration): A Population Study.” *The Lancet* 388 (10040): 131–157. doi: 10.1016/S0140-6736(16)00345-7 27108232

[CIT0003] Bakan, A. B., and E. Dua. 2014. *Theorizing Anti-Racism. Linkages in Marxims and Critical Race Theories*. Toronto: University of Toronto Press.

[CIT0004] Berrio Palomo, L. 2014. “Trayectorias reproductivas y practicas de attencion en la salud maternal entre mujeres indigenas de la costa chica de guerrero.” In *Trayetorias reproductivas, atención obstétrica y morbimortalidad maternal en México*, edited by A. Sánchez Bringas, 211–244. Mexico: UAM Mexico.

[CIT0005] Briggs, C. 1986. *Learning How to Ask*. Cambridge, UK: Cambridge University Press.

[CIT0006] Chopel, A. 2014. “Reproductive Health in Indigenous Chihuahua: Giving Birth ‘Alone Like the Goat.’” *Ethnicity and Health* 19 (3): 270–296. doi: 10.1080/13557858.2013.771150 23444879

[CIT0007] Colombara, C., B. Hernández, A. Schaefer, N. Zyznieuski, M. F. Bryant, S. Desai, M. Gagnier, et al. 2016. “Institutional Delivery and Satisfaction among Indigenous and Poor Women in Guatemala, Mexico, and Panama.” *PLoS ONE* 11 (4): e0154388. doi: 10.1371/journal.pone.0154388 27120070 PMC4847770

[CIT0008] CONAPO (National Population Council). 2005. Data on Child Mortality by Municipality. Accessed September 29, 2016. http://www.conapo.gob.mx/es/CONAPO/Base_de_datos.

[CIT0009] Connell, R. W. 1987. *Gender and Power*. Cambridge, UK: Polity press.

[CIT0010] Farmer, P. 2005. *Pathologies of Power. Health, Human Rights and the New War on the Poor*. Berkeley, CA: University of California Press.

[CIT0011] Farmer, P. E., B. Nizeye, S. Stulac, and S. Keshavjee. 2006. “Structural Violence and Clinical Medicine.” *PLoS Medicine* 3 (10): 1686–1991. doi: 10.1371/journal.pmed.0030449 PMC1621099 17076568

[CIT0012] Freyermuth Enciso, G., and H. Argüello Avendaño. 2010. “La muerte prematura de mujeres en Los Altos de Chiapas. Un análisis desde la violencia.” *Revista Pueblos y Fronteras Digital* 5 (10): 181–216. doi: 10.22201/cimsur.18704115e.2010.10.150

[CIT0013] Gamlin, J. 2016. “Huichol Migrant Laborers and Pesticides: Structural Violence and Cultural Confounders.” *Medical Anthropology Quarterly* 30 (3): 303–320. doi: 10.1111/maq.12249 26818491 PMC5066708

[CIT0014] González Montes, S., and R. Valdez Santiago. 2008. “Violencia hacia las mujeres en ocho regiones indígenas de México: notas metodológicas en torno a la Encuesta Nacional sobre Salud y Derechos de las Mujeres Indígenas (ENSADEMI).” *Estudios Sociológicos* 26 (77): 435–450.

[CIT0015] Gracey, M., and M. King. 2009. “Indigenous Health Part 1: Determinants and Disease Patterns.” *The Lancet* 374 (9683): 65–75. doi: 10.1016/S0140-6736(09)60914-4 19577695

[CIT0016] Griffiths, K., C. Coleman, V. Lee, and R. Madden. 2016. “How Colonisation Determines Social Justice and Indigenous Health—A Review of the Literature.” *Journal of Population Research* 33: 9–30. doi: 10.1007/s12546-016-9164-1

[CIT0017] Hall, G., and H. Patrinos. 2005. *Indigenous Peoples, Poverty and Human Development in Latin America: 1994-2004*. Washington, DC: World Bank.

[CIT0018] Hernández, B., D. Ramírez-Villalobos, M. B. Duarte, A. Corcho, G. Villareal, A. Jiménez, and L. M. Torres. 2012. “Subregistro de defunciones de menores y certificación de nacimiento en una muestra representativa de los 101 municipios con más bajo índice de desarrollo humano en México 2012.” *Salud Pública Mexico* 54 (4): 393–400. doi: 10.1590/S0036-36342012000400009 22832831

[CIT0019] Holmes, S. M. 2012. “The Clinical Gaze in the Practice of Migrant Health: Mexican Migrants in the United States.” *Social Science &amp; Medicine* 74 (6): 873–881. doi: 10.1016/j.socscimed.2011.06.067 21992736

[CIT0020] Holmes, S., and H. Castañeda. 2014. *Ethnographic Studies. In Migration and Health Research Methdologies: A Handbook for the Study of Migrant Populations*. Berkeley, CA: University of California Press.

[CIT0021] INEGI. 2015a. “Tabulados de la Encuesta Intercensal 2015.” Accessed September 12, 2016. En: http://www3.inegi.org.mx/sistemas/tabuladosbasicos/default.aspx?c=33725&s=est.

[CIT0022] INEGI. 2015b. “México en cifras. Información nacional, por entidad federativa y municipios. 286 indicadores principales del Banco de Información.” Accessed September 19, 2016. I-NEGI, en: http://www3.inegi.org.mx/sistemas/mexicocifras/.

[CIT0023] King, M., A. Smith, and M. Gracey. 2009. “Indigenous Health Part 2: The Underlying Causes of the Health Gap.” *The Lancet* 374 (9683): 76–85. doi: 10.1016/S0140-6736(09)60827-8 19577696

[CIT0024] Kirmayer, L., and G. Brass. 2016. “Addressing Global Health Disparities among Indigenous Peoples.” *The Lancet* 388 (10040): 105–106. doi: 10.1016/S0140-6736(16)30194-5 27108233

[CIT0025] Lee, A. C. C., S. Cousens, G. L. Darmstadt, H. Blencowe, R. Pattison, N. Moran, G. J. Hofmeyr, R. Haws, S. Zulfiquar Butta, and J. Lawn. 2011. “Care during Labor and Birth for the Prevention of Intrapartum-Related Neonatal Deaths: A Systematic Review and Delphi Estimation of Mortality Effect.” *BMC Public Health* 11 (Suppl. 3): S10. doi: 10.1186/1471-2458-11-S3-S10 PMC3231883 21501427

[CIT0026] Leyva-Flores, R., C. Infante-Xibille, J. P. Gutiérrez, and F. Quintino Peréz. 2013. “Inequidad persistente en salud y acceso a los servicios para los pueblos indígenas de México, 2006-2012.” *Salud Pública de México* 55 (2): s123–s128. doi: 10.21149/spm.v55s2.5107 24626687

[CIT0027] Mokdad, A., K. Ellicott, P. Paola Zúñiga-Brenes, D. Ríos-Zertuche, E. B. Palmisano, E. Alfaro Porras, B. W. Anderson, N. Borgo, S. Desai, and M. C. Gagnier. 2015. “Salud Mesoamérica 2015 Initiative: Design, Implementation, and Baseline Findings.” *Population Health Metrics* 13 (3): 1–16. 25685074 10.1186/s12963-015-0034-4 PMC4327957

[CIT0028] Montenegro, R., and C. Stephens. 2006. “Indigenous Health 2. Indigenous Health in Latin America and the Caribbean.” *The Lancet* 367: 1859–1869. doi: 10.1016/S0140-6736(06)68808-9 16753489

[CIT0029] Paradies, Y. 2016. “Colonisation, Racism and Indigenous Health.” *Journal of Population Research* 33: 83–96. doi: 10.1007/s12546-016-9159-y

[CIT0030] Pfeiffer, J., and M. Nichter. 2008. “What Can Critical Medical Anthropology Contribute to Global Health?” *Medical Anthropology Quarterly* 22 (4): 410–415. doi: 10.1111/j.1548-1387.2008.00041.x 19189725

[CIT0031] Risman, B. 2004. “Gender as a Social Structure. Theory Wrestling with Activism.” *Gender and Society* 18 (4): 429–450. doi: 10.1177/0891243204265349

[CIT0032] Scheper-Hughes, N. 1992. *Death without Weeping*. Berkeley, CA: University of California Press.

[CIT0033] Sesia, P., A. Zentella, K. Ruiz, et al. 2007. “Violencia y mortalidad materna en contextos indígenas de Oaxaca: una mirada etnográfica.” *GenEros* 4 (1): 53–83.

[CIT0034] Smith-Oka, V. 2009. “Unintended Consequences: Exploring the Tensions between Development Programs and Indigenous Women in Mexico in the Context of Reproductive Health.” *Social Science and Medicine* 68: 2069–2077. doi: 10.1016/j.socscimed.2009.03.026 19362404

[CIT0035] Stephens, C., J. Porter, C. Nettleton, and R. Willis. 2006. “Disappearing, Displaced and Undervalued: A Call to Action for Indigenous Health Worldwide.” *The Lancet* 367: 2019–2028. doi: 10.1016/S0140-6736(06)68892-2 16782493

[CIT0036] Tomé, P., H. Reyes, C. Piña, et al. 1997. “Características asociadas al subregistro de muerte en niños del estado de Guerrero, México.” *Salud Pública Mexico* 39: 523–529. doi: 10.1590/S0036-36341997000600005 9477734

[CIT0037] Tong, A., P. Sainsbury, and J. Craig. 2007. “Consolidated Criteria for Reporting Qualitative Research (COREQ): A 32-Item Checklist for Interviews and Focus Groups.” *International Journal for Quality in Health Care* 19 (6): 349–357. doi: 10.1093/intqhc/mzm042 17872937

[CIT0038] UNDP. 2010. Informe sobre desarrollo humano de los pueblos indígenas de México. El reto de la desigualdad de oportunidades. United Nations Development Programme. PNUD/ CDI-Comisión Nacional para el Desarollo de los Pueblos Indígenas 2010, México. Accessed September 29, 2016. http://hdr.undp.org/sites/default/files/mexico_nhdr_2010.pdf.

[CIT0039] Valeggia, C., and J. Snodgrass. 2015. “Health of Indigenous Peoples.” *Annual Review of Anthropology* 44: 117–135. doi: 10.1146/annurev-anthro-102214-013831

[CIT0040] Wang, H., C. A. Liddel, M. Coates, M. D. Mooney, C. E. Levitz, A. E. Schumacher, H. Apfel, et al. 2014. “Global, Regional, and National Levels of Neonatal, Infant, and under-5 Mortality during 1990–2013: A Systematic Analysis for the Global Burden of Disease Study 2013.” *The Lancet* 384: 957–979. doi: 10.1016/S0140-6736(14)60497-9 PMC4165626 24797572

[CIT0041] WHO. 2013. http://www.who.int/maternal_child_adolescent/epidemiology/profiles/neonatal_child/mex.

[CIT0042] WHO. 2016. “Health in Indigenous Communities.” Note 326. Accessed December 13, 2016. http://www.who.int/mediacentre/factsheets/fs326/es/.

[CIT0043] Zacher Dixon, L. 2015. “Obstetrics in a Time of Violence: Mexican Midwives Critique Routine Hospital Practices.” *Medical Anthropology Quarterly* 29 (4): 437–454. doi: 10.1111/maq.12174 25411151

